# Widespread exfoliative dermatitis in systemic lupus erythematosus (SLE): a rare clinical image

**DOI:** 10.11604/pamj.2026.53.72.50490

**Published:** 2026-02-10

**Authors:** Garima Gupta, Shweta Parwe

**Affiliations:** 1 Department of Panchakarma, Mahatma Gandhi Ayurveda College Hospital and Research Centre, Salod (Hirapur), Datta Meghe Institute of Higher Education and Research, Wardha, Maharashtra, India

**Keywords:** Exfoliative dermatitis, systemic lupus erythematosus, chronic erythematous scaling plaques, cutaneous lupus

## Image in medicine

An adult female aged 55 years presented with a progressively worsening exfoliative eruption involving the face, scalp, trunk, and upper back. She reported generalised dryness, burning, and photosensitivity over several weeks. Clinical examination showed diffuse erythema, scaling, and patchy alopecia over the frontal scalp. The face and anterior neck demonstrated erythematous plaques with superficial exfoliation. Multiple scaly, erythematous lesions were seen across the upper back, extending over the posterior trunk and shoulders in a photo-exposed pattern. Based on the morphology, chronicity, and photosensitive distribution, exfoliative dermatitis secondary to cutaneous involvement of systemic lupus erythematosus (SLE) was suspected. Differential diagnoses considered included erythrodermic psoriasis and chronic eczematous dermatitis. A laboratory investigation, including antinuclear antibody testing and systemic evaluation, was recommended. Supportive Ayurvedic management was initiated with Arogyavardhini Vati and Gandhaka Rasayana (250 mg each, twice daily), along with a mild ghrita-based emollient for local application and strict photoprotection.

**Figure 1:**
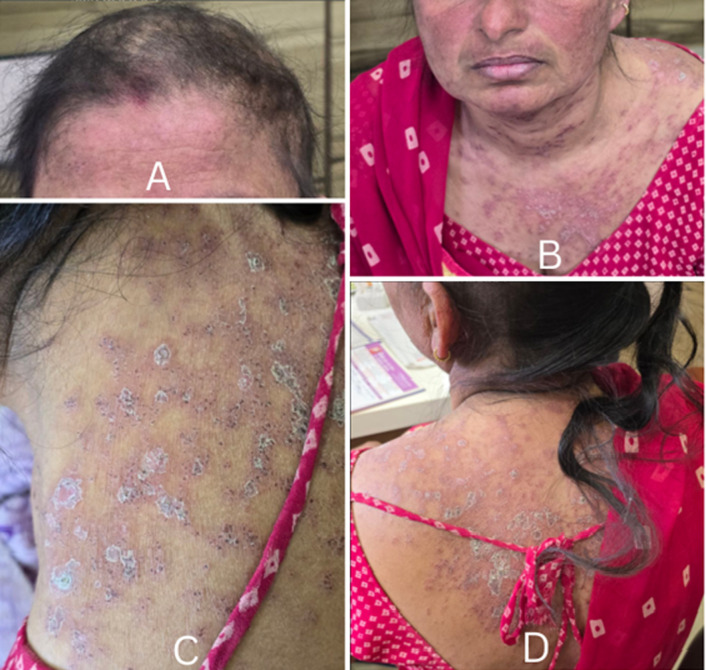
A) diffuse erythema, scaling, and patchy alopecia over the frontal scalp; B) facial erythema with superficial exfoliation involving the cheeks, chin, and neck; C) scaly erythematous plaques with surface crusting over the upper back; D) widespread exfoliative dermatitis over the posterior trunk and shoulders in a photo-exposed distribution

